# Comparing benzodiazepine-ketamine and benzodiazepine-fentanyl sedation in phacoemulsification: A double-blind crossover non-inferiority clinical trial (BEKEF study)

**DOI:** 10.1016/j.aopr.2025.04.001

**Published:** 2025-04-03

**Authors:** Adriano Cypriano Faneli, Ricardo Danilo Chagas Oliveira, Pablo Amado, Eduardo F. Marback, Rodrigo Amaral Torres, Juliana Fernandes Marback, Larrie Laporte, Caio Vinicius Saito Regatieri, Cristina Muccioli

**Affiliations:** a Bahiana School of Medicine and Public Health, Salvador, Brazil; b HOBrasil, Salvador, Brazil; c Federal University of Bahia, Ophthalmology Department, Salvador, Brazil; d Federal University of São Paulo, Ophthalmology Department, São Paulo, Brazil

**Keywords:** Cataract surgery, Sedation, Ketamine, Fentanyl, Phacoemulsification

## Abstract

**Background:**

Topical anesthesia for cataract surgery often requires adjunctive sedation to manage intraoperative discomfort and improve patient cooperation. Ketamine and fentanyl, combined with benzodiazepines, are commonly used sedation regimens, but their comparative safety and efficacy in the cataract surgery context remain underexplored. This study aimed to evaluate whether ketamine combined with midazolam is non-inferior to fentanyl combined with midazolam for sedation during phacoemulsification, with a non-inferiority margin of 10%.

**Methods:**

This prospective, double-blind, crossover, non-inferiority trial randomized 75 patients to receive both sedation regimens for bilateral phacoemulsification. A 15-day washout period was implemented between surgeries. Adequate sedation was defined as a Ramsay Sedation Scale score of 2–3. The primary outcome was sedation adequacy, with secondary outcomes including patient and surgeon satisfaction, surgical metrics, and complications.

**Results:**

Of the 75 randomized patients, 65 (130 eyes) completed the study. Adequate sedation was achieved in 86.2% of cases with ketamine and 89.2% with fentanyl, with a within-participant difference of 3.1% (95% CI: −2.3%–5.3%), confirming non-inferiority. Patient satisfaction scores were similarly high between regimens (ketamine: 4.87 ​± ​0.36; fentanyl: 4.91 ​± ​0.28; *P* ​= ​0.45). Complications were infrequent, with two cases of nausea and two of bradycardia in the fentanyl group and one case of nausea and two of hypertension in the ketamine group.

**Conclusions:**

Ketamine combined with midazolam is a safe and effective alternative to fentanyl-based sedation for cataract surgery, providing comparable sedation quality and satisfaction. These findings support ketamine's use in cataract surgery.

## 1 Introduction

Cataracts are a leading cause of vision impairment and blindness worldwide, significantly impacting the quality of life for millions of individuals annually.^1^ Phacoemulsification has become the gold-standard surgical technique for cataract extraction due to its minimally invasive nature and faster recovery times.^2^^,^^3^ While advancements in surgical methods have reduced procedural risks, the reliance on topical anesthesia has introduced challenges in managing intraoperative pain, anxiety, and patient compliance.^4^, ^5^, ^6^

Topical anesthesia, widely adopted for phacoemulsification, offers reduced complication rates compared to previously used techniques, such as retrobulbar or peribulbar blocks.^7^ However, its limited efficacy in deep-tissue analgesia often necessitates adjunctive sedation to ensure patient comfort and surgeon satisfaction.^3^, ^4^, ^5^ Effective sedation alleviates patient anxiety and procedural discomfort and optimizes surgical outcomes by minimizing movement and enhancing cooperation during surgery.^8^^,^^9^

Among sedative agents, fentanyl and ketamine are frequently used due to their pharmacodynamic profiles.^10^ Fentanyl, a potent opioid analgesic, provides rapid onset and reliable sedation but carries risks of respiratory depression and nausea.^10^ Ketamine, a dissociative anesthetic, maintains cardiovascular stability and respiratory function but may cause hypertension, tachycardia, and psychotropic side effects.^10^ The combination of these agents with benzodiazepines has demonstrated synergistic effects, improving sedation quality while potentially reducing adverse outcomes.^11^^,^^12^

Despite their extensive use in areas outside of ophthalmology, evidence of the combination of ketamine and benzodiazepines in cataract surgery is lacking, primarily due to the risk of psychomimetic effects associated with high doses of ketamine, which has led to resistance against its use in cataract surgery^10^ Most studies have focused on individual drugs or different combinations, leaving a significant gap in the literature regarding the relative safety, efficacy, and satisfaction of both patients and surgeons with the use of ketamine combined with benzodiazepines.^12^, ^13^, ^14^ To address this gap, we designed a double-blind, crossover noninferiority trial to evaluate the sedative effectiveness and safety profile of ketamine combined with benzodiazepines in comparison to an established anesthetic regimen, fentanyl combined with benzodiazepines.^12^^,^^15^^,^^16^ Our primary hypothesis is that ketamine will be non-inferior to fentanyl in achieving adequate sedation during surgery while providing comparable levels of patient and surgeon satisfaction. The non-inferiority margin was set at 10%, based on clinical relevance and guidelines for non-inferiority margins.^17^ This study aims to improve clinical decision-making in the perioperative management of cataract surgery by generating robust evidence on the ketamine with benzodiazepine sedation regimen.

## 2 Materials and methods

This study was a prospective, randomized, double-blind, crossover, non-inferiority clinical trial to compare two intravenous sedation regimens during cataract surgery. Each participant received both regimens: ketamine combined with midazolam for one eye and fentanyl combined with midazolam for the other eye. The order of interventions was randomized in a 1:1 ratio. A washout period of at least 15 days between surgeries was implemented to minimize potential carryover effects. The crossover design was chosen to reduce interpatient variability, with each participant as their control. The non-inferiority margin for the primary outcome was set at 10%. No significant changes to the protocol or eligibility criteria were made after the trial commenced. This study adhered to the CONSORT statement for randomized crossover trials and the CONSORT statement for noninferiority trials.^18^^,^^19^

Patients aged 18 years or older, classified as American Society of Anesthesiologists (ASA) physical status I–II, and scheduled for bilateral phacoemulsification were included. Patients with chronic pain syndromes, hypersensitivity to study medications, significant communication difficulties, or ASA classification ​> ​III were excluded. All procedures occurred at HOBrasil, a private ophthalmological hospital in Salvador, Bahia, Brazil, between October and December 2024. The trial is registered with the Brazilian Registry of Clinical Trials under the identifier RBR-7c4c5jx.

Sedation regimens were administered intravenously. Participants in the ketamine group received ketamine (0.14 ​mg/kg) combined with midazolam (0.029 ​mg/kg), while those in the fentanyl group received fentanyl (0.71 mcg/kg) combined with midazolam (0.029 ​mg/kg).^10^^,^^20^ Oxygen was provided at a flow rate of 4 ​L/min via nasal cannula, and standard monitoring included ECG, pulse oximetry, and noninvasive blood pressure. Sedation levels were maintained to ensure comfort while preserving responsiveness, as defined by a Ramsay Sedation Scale (RSS) which consists of six criteria: a score of 1 indicates a patient who is awake, anxious, agitated, or restless; a score of 2 indicates a patient who is awake, cooperative, oriented, and tranquil; a score of 3 indicates a patient who is drowsy with response to commands; a score of 4 indicates a patient who is asleep but shows a brisk response to glabellar tap or loud auditory stimuli; a score of 5 indicates a patient who is asleep and sluggish to respond to stimuli; and a score of 6 indicates no response to firm nail-bed pressure or other noxious stimuli.^21^ The sedatives were prepared in identical syringes, ensuring similarity between regimens and maintaining blinding for participants, surgeons, and outcome assessors.

The primary outcome was sedation adequacy during surgery, assessed intraoperatively using the RSS. Sedation was considered adequate if participants achieved an intraoperative RSS score of 2 or 3. Noninferiority was defined as no more than a 10% reduction in the proportion of participants achieving adequate sedation with ketamine compared to fentanyl. Secondary outcomes included patient satisfaction during the procedure, which was assessed using a Likert scale ranging from 1 (very dissatisfied) to 5 (very satisfied). After the second surgery, participants were asked to indicate which sedation regimen they preferred. Intraoperative metrics were evaluated, such as surgical difficulty measured by the SURG-TLX scale and surgical duration recorded in minutes. Anesthesia-related complications, such as bradycardia, hypoxemia, nausea, and vomiting, were also documented. Data were collected preoperatively, intraoperatively, and postoperatively for each surgery. If further sedation was required, a rescue dose of 5 ​mg of ketamine was administered to the Ketamine group and 50 mcg of fentanyl to the Fentanyl group.

The sample size was determined based on an expected sedation success rate of 87% for fentanyl and 82% for ketamine. Assuming a noninferiority margin of 10% and a power of 80%, 48 eyes per group were required. This calculation accounted for within-participant variability to ensure adequate power for detecting noninferiority. No interim analyses were planned, and no formal stopping guidelines were defined.

Randomization was performed using computer-generated sequences stratified in blocks of four. The allocation sequence was managed using REDCap software to ensure concealment until the assignment.^22^^,^^23^ The anesthesiologist administering the sedation was aware of the allocation but was not involved in outcome assessments. Patients and surgeons remained blinded throughout the study.

Statistical analyses were conducted using Stata Statistical Software Release 18 (StataCorp LLC). Within-participant comparisons were employed for the crossover design. For the primary outcome, non-inferiority was tested using a two-sided 95% confidence interval approach, with sedation adequacy compared between regimens. Secondary outcomes were analyzed using a paired *t*-test for continuous variables and a chi-square test for categorical variables. Due to the crossover design, per protocol analysis was implemented.

The study was conducted in accordance with the Declaration of Helsinki and approved by the Institutional Review Board of Hospital Oftalmológico de Brasília (protocol 77052824.9.0000.5667). All participants provided written informed consent before enrollment.

## 3 Results

**Fig. 1:**
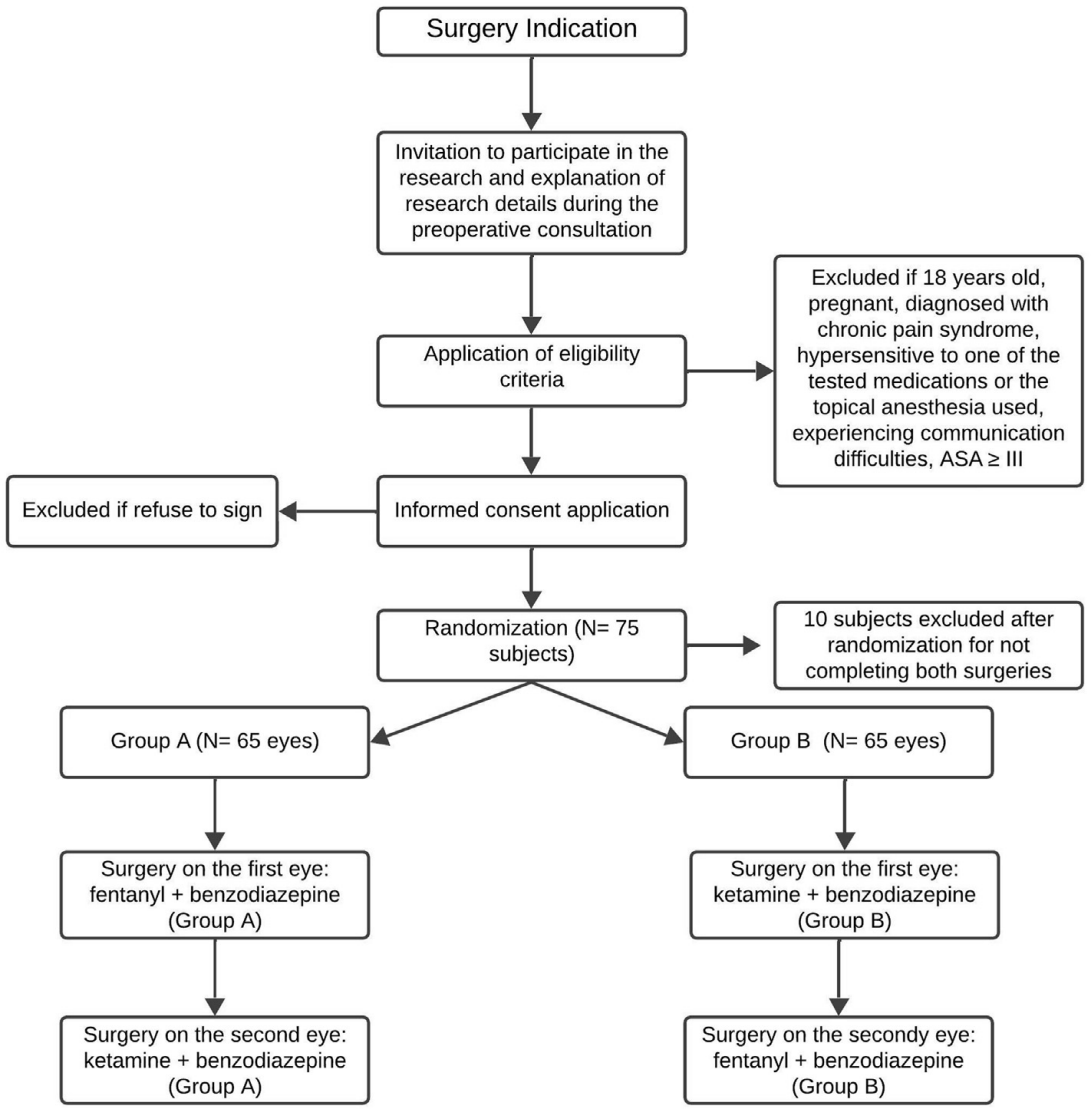
*Flowchart of Study Participants* This figure illustrates the flow of participants through each stage of the study. A total of 75 patients were initially randomized to receive both sedation regimens in a crossover design. Ten participants were excluded after randomization for not completing both planned surgeries. The remaining 65 patients (130 eyes) were distributed into two groups: Group A (65 eyes) and Group B (65 eyes). Group A received fentanyl with benzodiazepine for the first eye and ketamine with benzodiazepine for the second eye. Group B received ketamine with benzodiazepine for the first eye and fentanyl with benzodiazepine for the second eye. No patients were excluded before randomization.

In a crossover design, 75 patients were initially randomized to receive both sedation regimens. Of these, ten patients were excluded after randomization for not completing one of the two planned surgeries, resulting in 65 patients (130 eyes) completing the study. The flow of participants through each stage of the study, including exclusions, is illustrated in Fig. 1.

The trial was completed as planned with no interruptions. Participants were between 54 and 87 years old, with a mean age of 69.2 years (SD ​± ​6.9). The majority (61%) were female. Regarding racial distribution, 34 participants were White, 7 Black, and 24 identified as Other. Systemic comorbidities were present in 44 participants, with hypertension in 26, type 2 diabetes mellitus in 11, and other conditions in 7. Twenty-one participants had no systemic comorbidities. 43 participants were classified as ASA II, and 22 as ASA I. Table 1 presents a detailed demographic and clinical characteristics summary.

**Table 1:** Sample characteristics.

Mean age (years, SD)	69.2 (6.9)
Female, n (%)	40 (61%)
Male, n (%)	25 (39%)
Racial distribution, n (%)	
White	34 (52%)
Black	7 (11%)
Other	24 (37%)
Systemic comorbidities, n (%)	44 (68%)
Hypertension	26 (40%)
Type 2 Diabetes Mellitus	11 (17%)
Other	7 (11%)
ASA classification, n (%)	
ASA I	22 (34%)
ASA II	43 (66%)

Adequate sedation (RSS 2–3) during surgery was achieved in 58 (89.2%) of eyes in the fentanyl group and 56 eyes (86.2%) in the ketamine group. The within-participant difference was 3.1% (95% CI: −2.3%–5.3%), within the predefined noninferiority margin of 10%, confirming the noninferiority of ketamine relative to fentanyl for sedation. Five eyes in the fentanyl group and eight eyes had an intraoperative RSS of 4. Two eyes in the fentanyl group and one eye in the ketamine group had an intraoperative RSS of 5. Five eyes in the ketamine group and two eyes in the fentanyl group required additional intraoperative sedation. Table 2 details these results, including the distribution of RSS scores and patient-reported satisfaction.

**Table 2:** Intraoperative sedation levels and patient regimen preference.

	Fentanyl (n ​= ​65)	Ketamine (n ​= ​65)
Adequate sedation (RSS 2–3), n (%)	58 (89.2%)	56 (86.2%)
RSS 4 intraoperative, n	5	8
RSS 5 intraoperative, n	2	1
Additional intraoperative sedation	2	5
Patient-reported satisfaction (Likert scale, mean ​± ​SD)	4.91 ​± ​0.28	4.87 ​± ​0.36
Patient preference, n (%)	36 (55.3%)	29 (44.6%)

No sedation-related complications or surgical complications occurred in either group. Anesthesia-related complications were infrequent but included three events in the ketamine group (one case of nausea and two cases of hypertension) and four events in the fentanyl group (two cases of bradycardia and two cases of nausea). Table 3 summarizes these complications.

**Table 3:** Complications distribution.

Complication	Fentanyl (n ​= ​65)	Ketamine (n ​= ​65)
Anesthesia-related complications		
Nausea, n	2	1
Hypertension, n	0	2
Bradycardia, n	2	0
Surgical complications	0	0
Sedation-related complications	0	0

Patient-reported satisfaction with sedation was similar between regimens. The ketamine group achieved an average satisfaction score of 4.87 (SD ​± ​0.36), and the fentanyl group averaged 4.91 (SD ​± ​0.28). After the second surgery, 36 participants preferred the fentanyl sedation, while 29 preferred ketamine. These data are summarized in Table 2. Vital signs remained stable across all stages of the procedures in both groups. Table 4 presents the oxygen saturation, heart rate, systolic blood pressure, and diastolic blood pressure data for both groups.

**Table 4:** Vital signs across procedural stages.

Stage	Fentanyl (mean ​± ​SD)	Ketamine (mean ​± ​SD)
Oxygen Saturation (%)		
Preoperative	98.5 ​± ​1.1	98.5 ​± ​1.2
Intraoperative	99.3 ​± ​0.9	99.2 ​± ​1.2
Postoperative	97.2 ​± ​2.4	97.5 ​± ​3.1
Heart Rate (bpm)		
Preoperative	71.5 ​± ​10.0	70.6 ​± ​11.3
Intraoperative	69.7 ​± ​11.0	69.8 ​± ​10.5
Postoperative	68.5 ​± ​10.0	67.8 ​± ​10.8
Systolic Blood Pressure (mmHg)		
Preoperative	146.2 ​± ​21.0	142.3 ​± ​17.9
Intraoperative	140.9 ​± ​39.8	144.3 ​± ​38.8
Postoperative	136.8 ​± ​21.6	142.1 ​± ​19.7
Diastolic Blood Pressure (mmHg)		
Preoperative	81.4 ​± ​11.6	82.0 ​± ​11.9
Intraoperative	75.7 ​± ​13.4	79.5 ​± ​12.4
Postoperative	78.2 ​± ​14.7	79.6 ​± ​12.0

Intraoperative metrics showed comparable surgical difficulty scores, with a mean SURG-TLX score of 12.1 ​± ​12.0 (range, 6–60) for the ketamine group and 11.5 ​± ​11.8 (range, 6–65) for the fentanyl group (*P* ​= ​0.34). Surgical duration ranged from 2 to 20 ​min in the ketamine group (mean, 10.8 ​min, SD ​± ​2.4) and from 8 to 20 ​min in the fentanyl group (mean, 11.38 ​min, SD ​± ​2.2) (*P* ​= ​0.90). These metrics are detailed in Table 5.

**Table 5:** Intraoperative difficulty metrics.

	Fentanyl (mean ​± ​SD) [range]	Ketamine (mean ​± ​SD) [range]
Surgical difficulty (SURG-TLX score)	11.5 ​± ​11.8 [6–65]	12.1 ​± ​12.0 [6–60]
Surgical duration (minutes)	11.38 ​± ​2.2 [8, 9, 10, 11, 12, 13, 14, 15, 16, 17, 18, 19, 20]	10.8 ​± ​2.4 [2, 3, 4, 5, 6, 7, 8, 9, 10, 11, 12, 13, 14, 15, 16, 17, 18, 19, 20]

## 4 Discussion

This study demonstrated the non-inferiority of ketamine combined with midazolam relative to fentanyl combined with midazolam for sedation during phacoemulsification cataract surgery. Adequate sedation was achieved in 86.2% of cases in the ketamine group and 89.2% in the fentanyl group, with a within-participant difference of 3.1% (95% CI: −2.3%–5.3%), well within the predefined non-inferiority margin of 10%. Patient satisfaction was similarly high in both sedation regimens (*P* ​= ​0.45). There was no significant difference between the patients' preferences for the two regimens (*P* ​= ​0.60). Surgical difficulty, assessed via SURG-TLX score (mean 12.1 ​± ​12.0 for ketamine and 11.5 ​± ​11.8 for fentanyl, *P* ​= ​0.34), and surgical duration were comparable between groups (mean 10.8 ​± ​2.4 ​min for ketamine and 11.38 ​± ​2.2 ​min for fentanyl, *P* ​= ​0.90), further validating ketamine's viability as a sedation option for cataract surgery.

Furthermore, the non-existent psychotropic side effects observed in our study align with previous research suggesting that doses of at least 1 ​mg/kg are necessary to induce significant psychotropic effects.^14^ This finding reinforces the safety of low-dose ketamine, particularly when combined with benzodiazepines, which are known to mitigate the risk of psychotropic phenomena.^24^

Hemodynamic stability was observed in both groups, with ketamine associated with transient hypertension in two cases and fentanyl demonstrating two cases of bradycardia. These phenomena are related to the pharmacodynamic properties of the respective agents. Ketamine exerts its effects primarily through antagonism of the N-methyl-D-aspartate receptors in the central nervous system.^14^ This interaction activates the sympathetic nervous system, which explains the occasional hypertensive episodes observed in our study.^14^ In contrast, fentanyl predominantly acts as a potent μ-opioid receptor agonist, influencing vagal tone at the cardiac level and thereby contributing to bradycardia.^14^

Nausea was present in both groups, with two cases observed in the fentanyl group and one in the ketamine group. Fentanyl, as a μ-opioid receptor agonist, stimulates the chemoreceptor trigger zone in the medulla, which is directly associated with nausea and vomiting.^25^ Ketamine, though less commonly associated with nausea, may influence the vestibular system and neurotransmitter pathways such as serotonin and noradrenaline, potentially contributing to this side effect.^14^^,^^26^ These effects could be mitigated by the preoperative administration of antiemetic agents, which was not implemented in our study to allow an unbiased observation of nausea frequency between the two sedation regimens.^27^

Previous studies have highlighted the effectiveness of ketamine and midazolam in different contexts. Cheuk et al. demonstrated their rapid and effective sedation in pediatric procedures, with dose-dependent and manageable adverse effects.^28^ Similarly, Chudnofsky et al. confirmed the safety and efficacy of this combination in adult emergency settings, noting minimal respiratory compromise and high patient satisfaction.^29^ Our study extends these findings to cataract surgery, confirming the combination's safety, hemodynamic stability, and quality of sedation. These results validate ketamine combined with midazolam as a safe and effective alternative to fentanyl-based regimens, particularly in patients with contraindications to opioids or at risk for opioid-related side effects. Moreover, Heidari et al. also showed that propofol–ketamine provided effective sedation and stable hemodynamics during cataract surgery, reinforcing our findings on ketamine's safety profile.^30^

This study has limitations. First, the possibility of carryover effects in the crossover design cannot be excluded despite implementing a 15-day washout period. Additionally, the study population was limited to patients classified as ASA I–II, which restricts the generalizability of these findings to individuals with more complex medical conditions. Furthermore, while sedation adequacy and patient and surgeon satisfaction were assessed, the reliance on subjective measures may have introduced bias. Despite these limitations, the study's robust design, including the use of within-patient comparisons and adherence to the previously published protocol, enhances the reliability of its findings.^31^

## 5 Conclusions

This study demonstrates that ketamine combined with midazolam is a non-inferior, safe, and effective alternative to fentanyl-based sedation during phacoemulsification cataract surgery. Comparable rates of sedation adequacy, high patient satisfaction, and a low incidence of complications were observed in both regimens. The hemodynamic stability and absence of significant psychotropic effects with ketamine further support its viability, especially for patients with contraindications to opioids. These findings provide robust evidence for incorporating ketamine into sedation protocols for cataract surgery.

## Study approval

The study adhered to the basic principles of the Declaration of Helsinki. Ethics committee approval was granted by the Ethics Committee) of Hospital Oftalmológico de Brasília (protocol code 77052824.9.0000.5667) on July 9th, ​2024.

## Authors contributions

All authors attest that they meet the current International Committee of Medical Journal Editors (ICMJE) criteria for Authorship. The authors confirm contribution to the paper as follows: Conception and design of study: AF, RO, PA, RT, JM, LL, CR, CM; Data collection: AF, PA, RO, EM; Analysis and interpretation of results: AF, CR, CM; Drafting the manuscript: AF, CR, CM; All authors reviewed the results and approved the final version of the manuscript.

## Funding

This research did not receive any specific grant from funding agencies in the public, commercial, or not-for-profit sectors.

## Declaration of competing interest

The authors declare that they have no known competing financial interests or personal relationships that could have appeared to influence the work reported in this paper.
